# Association of Polymorphisms of Genes Involved in Lipid Metabolism with Blood Pressure and Lipid Values in Mexican Hypertensive Individuals

**DOI:** 10.1155/2014/150358

**Published:** 2014-12-21

**Authors:** Blanca Estela Ríos-González, Bertha Ibarra-Cortés, Guadalupe Ramírez-López, José Sánchez-Corona, María Teresa Magaña-Torres

**Affiliations:** ^1^División de Genética, Centro de Investigación Biomédica de Occidente, Instituto Mexicano del Seguro Social, Sierra Mojada No. 800, Colonia Independencia, 44340 Guadalajara, JAL, Mexico; ^2^Centro Universitario de Ciencias de la Salud, Universidad de Guadalajara, Sierra Mojada No. 950, Colonia Independencia, 44348 Guadalajara, JAL, Mexico; ^3^Instituto de Genética Humana “Enrique Corona Rivera”, Centro Universitario de Ciencias de la Salud, Universidad de Guadalajara, Sierra Mojada No. 950, Colonia Independencia, 44348 Guadalajara, JAL, Mexico; ^4^Unidad de Investigación Epidemiológica y en Servicios de Salud del Adolescente, Instituto Mexicano del Seguro Social, Avenida Tonalá 121, 45400 Tonalá, JAL, Mexico; ^5^División de Medicina Molecular, Centro de Investigación Biomédica de Occidente, Instituto Mexicano del Seguro Social, Sierra Mojada No. 800, Colonia Independencia, 44340 Guadalajara, JAL, Mexico

## Abstract

Hypertension and dyslipidemia exhibit an important clinical relationship because an increase in blood lipids yields an increase in blood pressure (BP). We analyzed the associations of seven polymorphisms of genes involved in lipid metabolism (*APOA5* rs3135506, *APOB* rs1042031, *FABP2* rs1799883, *LDLR* rs5925, *LIPC* rs1800588, *LPL* rs328, and *MTTP* rs1800591) with blood pressure and lipid values in Mexican hypertensive (HT) patients. A total of 160 HT patients and 160 normotensive individuals were included. Genotyping was performed through PCR-RFLP, PCR-AIRS, and sequencing. The results showed significant associations in the HT group and HT subgroups classified as normolipemic and hyperlipemic. The alleles *FABP2* p.55T, *LIPC* −514T, and *MTTP* −493T were associated with elevated systolic BP. Five alleles were associated with lipids. *LPL* p.474X and *FABP2* p.55T were associated with decreased total cholesterol and LDL-C, respectively; *APOA5* p.19W with increased HDL-C; *APOA5* p.19W and *FABP2* p.55T with increased triglycerides; and *APOB* p.4181K and *LDLR* c.1959T with decreased triglycerides. The *APOB* p.E4181K polymorphism increases the risk for HT (OR = 1.85, 95% CI: 1.17–2.93; *P* = 0.001) under the dominant model. These findings indicate that polymorphisms of lipid metabolism genes modify systolic BP and lipid levels and may be important in the development of essential hypertension and dyslipidemia in Mexican HT patients.

## 1. Introduction

Hypertension and dyslipidemia are major global public health problems because they are highly prevalent, contribute to the development of cardiovascular disease, and are components of metabolic syndrome. In Mexico, 26.6% of the working-age population is hypertensive (HT), 48.7% exhibits high total cholesterol (TC) levels (≥200 mg/dL), and 57.3% displays high triglyceride (TG) concentrations (≥150 mg/dL) [1]. Essential hypertension (EH) is a multifactorial disease, whose development involves environmental and genetic factors. A significant percentage (30%–60%) of blood pressure (BP) variation is due to genetic factors, and at least 69 genes have been associated with EH [2].

A group of genes that have been less well studied but may play an important role in EH development are those encoding proteins involved in lipid metabolism. Hypertension and dyslipidemia show an important clinical relationship because an increase in nonesterified fatty acids (NEFAs) (lipoprotein metabolism products) increases BP, but, in normal conditions, the endocrine, sympathetic, and parasympathetic systems stabilize BP [3]. However, in the presence of chronic hyperlipidemia, such regulatory mechanisms are insufficient, and the sustained increase in NEFAs directly affects cardiac output and peripheral resistance, which are important hemodynamic variables that regulate BP. Cardiac output can be altered by a decrease in the sensitivity of baroreceptors, increased synthesis of catecholamine, and an increased heart rate [3, 4], while peripheral resistance may increase by alpha-1 adrenergic receptor hypersensitivity [5], endothelial dysfunction, and atherosclerotic plaque formation [6].

Considering the close relationship between BP and blood lipid levels, we selected seven polymorphisms of seven genes that have mainly been associated with cardiovascular disease, atherosclerosis, dyslipidemias, and obesity. (1)* APOA5* p.S19W (rs3135506): the APOAV protein plays an important role in triglyceride (TG) metabolism, and the p.19W allele is negatively correlated with the secretion of this protein [7] and has been associated with increased TG and high-density lipoprotein cholesterol (HDL-C) levels [8, 9]. (2)* APOB* p.E4181K (rs1042031): the APOB protein is the main apolipoprotein found in chylomicrons (CM) and low-density cholesterol lipoproteins (LDL-C). The p.4181K allele increases LDL-C catabolism; therefore, it is associated with low TC, LDL-C, and APOB protein levels [10]. (3)* FABP2 *p.A55T (rs1799883): the FABP2 protein is involved in fatty acid use in the intestine, and the p.55T allele shows a higher affinity for fatty acids; hence, it promotes TG-rich lipoprotein formation [11]. (4)* LDLR* c.1959C>T (rs5925): the LDL receptor is involved in cholesterol homeostasis through lipoprotein endocytosis, and the c.1959T allele is associated with low TC, LDL-C, HDL-C, and TG in different groups of individuals [12, 13]. (5)* LIPC* −514 C>T (rs1800588): the LIPC protein functions as both a lipase and ligand, and the −514T allele has been positively correlated with HDL-C levels and negatively correlated with LIPC activity [14]. (6)* LPL* p.S474X (rs328): the LPL protein hydrolyses TG from very low-density lipoprotein cholesterol (VLDL-C) and CM. The mutated allele increases this enzymatic activity; hence, it improves TG-rich lipoprotein depuration [15]. (7)* MTTP* −493 G>T (rs1800591): MTTP is involved in VLDL-C and CM assemblage and secretion, and the homozygous genotype −493 TT has been associated with low TC, LDL-C, APOB, and MTTP mRNA levels [16].

The aim of the current study was to analyze the relationship of the* APOA5 *p.S19W,* APOB* p.E4181K,* FABP2* p.A55T,* LDLR* c.1959C>T,* LIPC* −514 C>T,* LPL* p.S474X, and* MTTP* −493 G>T polymorphisms with blood pressure and lipid levels in Mexican HT patients and in subgroups classified according to the presentation of different dyslipidemias.

## 2. Materials and Methods

### 2.1. Study Population

A total of 716 unrelated individuals aged 19–81 years were recruited from Family Medicine Unit number 93 and the Centro de Investigación Biomédica de Occidente of the Instituto Mexicano del Seguro Social in the metropolitan area of Guadalajara, Jalisco, Mexico. Blood samples were drawn from each of the subjects after at least 12 hours of fasting and without drinking alcohol for the preceding 72 hours. Two tubes of blood were collected; the first was used for biochemical testing, and the second was used to extract DNA. Biochemical measurements were performed using enzymatic methods with commercial kits and a semiautomatic ALS 2000 spectrophotometer. Teco diagnostics (Anaheim, CA), Trinder GOD-POD, Spinreact (Esteve de Bas, Girona, Spain), and AccuBind kits (Lake Forest, CA) were employed to determine the lipid profile (TC, LDL-C (using the Friedewald formula), HDL-C, and TG), glucose, and insulin, respectively. Blood pressure was measured according to the criteria proposed by NOM-030-SSA2-2009 [17].

From the total subjects (*n* = 716), we selected 320 individuals, mainly considering their blood pressure values, personal clinical history, age, and sex. The subjects included in the study consisted of 160 HT patients with systolic blood pressure ≥140 mm Hg and/or diastolic blood pressure ≥90 mm Hg [17] (121 women and 39 men), with a mean age of 47 ± 10 years (range 23–69 years), and 160 normotensive subjects (NT) with systolic blood pressure < 140 mm Hg and diastolic blood pressure < 90 mm Hg [17] (114 women and 46 men), with a mean age of 47 ± 11 years (range 20–81 years). Both groups were paired by 10-year age range and by sex, and no significant differences were identified based on the age (*P* = 0.69) or sex (*P* = 0.69) variables. None of the included individuals fulfilled all of the criteria for metabolic syndrome. The protocol was designed based on the guidelines outlined in the Declaration of Helsinki. It was reviewed and approved by the institute's ethics committee. The participants were informed of the study objectives and signed a consent letter.

### 2.2. Molecular Analysis

DNA was extracted from peripheral blood using standard procedures. Polymorphism genotyping was performed by means of three techniques: (a) polymerase chain reaction-restriction fragment length polymorphism (PCR-RFLP) analysis, to identify the* APOB* p.E4181K,* FABP2* p.A55T,* LDLR* c.1959C>T, and* LPL* p.S474X sites; (b) artificial introduction of restriction sites (PCR-AIRS), to detect* APOA5* p.S19W and* MTTP* −493G>T; and (c) sequencing, for the* LIPC* −514C>T site. The primers used to identify the* MTTP* −493G>T polymorphism were designed with Oligo 6.0 software (forward 5′ CTA GTG TGC TAA TGA CAG ACA ATG C 3′ and reverse 5′ GGA TTT AAA TTT AAA CTG TTA ATT CAT ATC AC 3′). A 155 bp fragment was amplified and digested with the* Hph* I enzyme. The PCR mixture included 13.3 ng/*μ*L genomic DNA, 0.46 pg/*μ*L of each primer, 0.204 mM of each dNTP, 1.5 mM Mg_2_Cl, and 0.04 U/*μ*L Taq polymerase. The final reaction volume was 15 *μ*L. The amplification program was as follows: initial denaturation for 4 min at 95°C, followed by 30 cycles of denaturation at 95°C for 25 sec, annealing at 91°C for 40 sec, and extension at 72°C for 45 sec, with a final extension at 72°C for 7 min.

The PCR and amplification conditions for the remaining six sites were described elsewhere [18].* FABP2* p.A55T, which was previously identified through sequencing [18], was genotyped in this work using the restriction enzyme* Hha* I. The samples employed as digestion controls were confirmed through sequencing in an ABI PRISM 310 sequencer using the BigDye Terminator v3.1 kit and following the manufacturer's instructions.

### 2.3. Statistical Analyses

The genotypic and allelic frequencies for the seven polymorphisms were established through direct counting. The frequencies were compared among the groups (NT and HT) using the chi-square or Fisher exact test. The effect of the polymorphisms on BP and lipid profile levels was determined in the group of HT patients and the HT subgroups classified as normolipemic and hyperlipemic according to lipid profile. An analysis of variance (ANOVA) was performed for the genotypes, followed by post hoc tests (Bonferroni or Dunnett, in relation to variance homogeneity). The mean differences between alleles were calculated with Student's *t-*test. The Pearson (for quantitative and normally distributed variables) and Spearman (for qualitative variables) correlations and multiple linear regression models with genotypes and alleles were employed. Furthermore, to estimate the relationships of the seven polymorphisms with hypertension and different dyslipidemias, multiple logistic regression models were generated. A value of *P* < 0.05 was considered statistically significant. The analyses were performed using SPSS v.18.0.

## 3. Results

### 3.1. Genotypic and Allelic Frequencies

The genotype and allele frequency distributions of the seven polymorphisms are shown in Table 1. All three possible genotypes were observed at each site and exhibited heterogeneous frequencies. The frequencies of the mutant genotypes of* FABP2* p.A55T,* LDLR* c.1959C>T, and* LIPC* −514C>T were approximately 30% in the HT and NT groups. The four remaining polymorphisms displayed frequencies lower than 5%. In both of the studied groups, the mutant alleles of* LDLR* c.1959T and* LIPC* −514T were more frequent than the wild-type alleles; in contrast* LPL* p.474X mutant allele did not exceed a 10% frequency.

Comparison of the genotypic and allelic frequencies between the groups revealed statistically significant differences only for the* APOB* p.E4181K polymorphism (*P* = 0.01 and *P* = 0.04, resp.) due to a higher percentage of heterozygotes in the HT group (Table 1).

### 3.2. Effect of the Polymorphisms on the Lipid Profile and Blood Pressure of HT Patients

In the association analysis between the polymorphisms versus the variables, 70 patients from the total HT group receiving antihypertensive and/or hypolipidemic treatments were excluded.

Considering all of the HT patients not receiving drug treatment (*n* = 90), the following significant associations with TC and TG were observed. Subjects with the* LPL* p.474X allele showed lower TC concentrations (185.0 mg/dL) than the patients with the p.474S allele (203.0 mg/dL, *P* = 0.006), while individuals with the* FABP2 *p.55T allele exhibited higher TG concentrations (161.2 mg/dL) than the patients with the p.55A allele (131.2 mg/dL; *P* = 0.006). A significant multiple linear regression model with the* FABP2 *p.55T and* LDLR* c.1959T alleles to explain TG levels was established (Table 4).

In regard to blood pressure, subjects carrying the* LIPC *−514T allele showed higher SBP values (151.2 mm Hg) than patients with the −514C allele (146.2 mm Hg), *P* = 0.039.

### 3.3. Hypertensive Patients Classified as Normolipemic and Hyperlipemic: Effect of the Polymorphisms on the Lipid Profile and Blood Pressure

All of the studied polymorphisms are located in genes that modulate lipid metabolism and have been associated mainly with dyslipidemia and/or cardiovascular disease. Dyslipidemias distribution in HT patients is shown in Table 2 and the classification was done according the NOM-037-SSA2-2002 [19].

Association analysis was performed in two subgroups of hypertensive patients, normolipemic (without dyslipidemia) and hyperlipemic (with dyslipidemia) (Table 2). Normolipemic subgroup was integrated by 50 patients, with a mean age of 50 ± 11 years (range 30–69 years). Hyperlipemic subgroup was formed by 40 patients, with a mean age of 45 ± 11 years (23–69 years). Age variable was not significantly different between subgroups (*P* = 0.60).

Analysis of the two subgroups showed many significant results. However, we present only those for which a multiple linear regression model was generated and showed significant values for ANOVA, Student's *t*-test, or Spearman correlation.  Table 3 provides the ANOVA, Student's *t*-test, and Spearman correlation results; two ANOVA nonsignificant *P* values were included to indicate trends (*FABP2* p. 55T allele for TG and SBP with *P* = 0.09 and *P* = 0.08, resp.). The multiple linear regression models for each polymorphism and quantitative variable are presented in Table 4. To avoid duplicate data, the values of the association tests are not provided in the text and are only shown in tables.

*APOA5 p.S19W*. The p.19W allele was associated with significant increases in HDL-C and TG in normolipemic and hyperlipemic HT patients, respectively.

*APOB p.E4181K*. We detected one significant association in the normolipemic subgroup; the HT patients carrying the p.4181K allele showed a lower TG concentration than those bearing the p.4181E allele.

*FABP2 p.A55T*. This polymorphism exhibited an association with three variables. TG and SBP levels were increased significantly in presence of the mutated allele p.55T in hyperlipemic and normolipemic subgroups, respectively; while LDL-C values were lower in hyperlipemic patients with p.55T versus p.55A.

*LPL p.S474X C>G*. The p.474X allele was associated with lower TC levels in HT hyperlipemic patients.

*MTTP −493 G>T*. Hyperlipemic patients carrying the −493T allele showed statistically higher SBP levels than those with the −493G allele (Tables 3 and 4).

### 3.4. Multiple Linear Regression Models

In total, nine distinct multiple linear regression models with statistically significant differences were generated with genotypes and alleles data; in Table 4, the results obtained with the alleles are shown. An integrated model with two polymorphisms* FABP2* p.A55T and* LDLR* c.1959C>T, explaining TG levels, was observed in the total group of hypertensive (*n* = 90) and hyperlipemic subgroup. The eight remaining models included only one polymorphism. The TG variable presented a greater number of significant models (Table 4).

### 3.5. Logistic Regression Models

In this analysis, we observed two models. The first was observed for* APOB* p.E4181K, which increased the risk of developing essential hypertension under the dominant model (EK+KK v. EE) (OR = 1.85; 95% CI: 1.2–2.9 and *P* = 0.01). The second model showed that HT patients carrying the* APOA5* p.19W allele exhibit an increased risk of presenting mixed hyperlipidemia (OR = 8.8; 95% CI: 1.76–44.0; *P* = 0.02) under the recessive model (WW v. SW+SS).

## 4. Discussion

Essential hypertension is a multifactorial disease with a strong genetic component; therefore, genes corresponding to different metabolic routes have been explored. Because increased blood lipid levels are known to result in an increase in blood pressure, in the present work, we analyzed seven polymorphisms of genes involved in lipid metabolism in HT patients to examine their role in the development of essential hypertension and dyslipidemias.

It is important to note that when the association analysis was performed in the entire HT group, only a few significant results were observed. However, the analysis of the HT patients classified according to lipid levels revealed more significant associations between the polymorphisms and diverse variables. Studies examining these polymorphisms in different populations have revealed heterogeneous results. A possible explanation is that these differences occur because most of the authors did not analyze the data by separating individuals according to dyslipidemia.

### 4.1. Genotypic and Allelic Frequencies

In the genotypic and allelic frequency distribution analyses for the HT and NT groups, we observed significant differences for the polymorphism* APOB *p.E4181K, based on the presence of more heterozygotes in the HT group (41.9% versus 23.3%). Other studies have analyzed this polymorphism in relation to cardiovascular disease, but the authors did not observe significant differences in the genotype and/or allele distribution [20, 21]

We compared the results for the seven polymorphisms with the reported frequencies in the general Mexican population [18, 22]. The analysis showed similar genotypic frequency distributions for the general population and NT individuals (*P* > 0.05). However, we observed significant differences between the general population and the HT group for three polymorphisms: the* APOB* p.E4181K site (*P* = 0.008),* FABP2* p.A55T (*P* = 0.002), and the* LIPC* −514C>T polymorphism (*P* = 0.04). Only two of the seven analyzed polymorphisms have been studied in HT patients:* LDLR* c.1959C>T (Chinese population) [12] and* LPL* p.S474X (Chinese and Caucasian populations) [23]; similar to the results of the present study, the authors did not report significant differences in the genotypic and allelic frequency distribution.

### 4.2. Distribution of Dyslipidemias in HT Patients

In Mexico, dyslipidemias and essential hypertension are the most common risk factors for the development of cardiovascular disease in the general adult population [24, 25]. In this study more than 40% of the subjects exhibited some form of dyslipidemia (hypercholesterolemia or hypertriglyceridemia or mixed hyperlipidemia), which indicates a significant health problem. Various reports have revealed that one of the most frequent dyslipidemias in the Mexican population is hypoalphalipoproteinemia, and similar results were obtained in this study. Low HDL-C levels in HT patients have also been observed in other populations at frequencies higher than 30% [26]. Although a clinical finding of hypoalphalipoproteinemia does not represent a health problem by itself, it is an important risk factor for developing cardiovascular disease and metabolic syndrome when associated with high TG and TC levels. Furthermore, in patients with hypertension, several coincident biochemical alterations can increase cardiovascular risk and complicate hypertension management.

### 4.3. Effect of the Polymorphisms on the Lipid Profile and Blood Pressure of Hypertensive Patients and Normolipemic and Hyperlipemic Subgroups

*APOA5 p.S19W*. The APOAV protein is a component of the lipoproteins HDL-C, VLDL-C, and CM; it activates LPL for efficient TG lipolysis. In this study, the p.19W allele was associated with increased HDL-C in normolipemic HT patients (Tables 3 and 4). This association between high HDL-C levels and the p.19W allele has been reported in healthy subjects from Puerto Rico [8].

High TG levels associated with the p.19W allele were observed in hyperlipemic HT patients. Similar results have been detected in young, healthy Caucasian males [9], Spanish subjects in the ICARIA project [27], and Caucasian children 6–8 years old [28]. This association was also reflected in the logistic regression analysis because, in the HT group, the p.19W allele (under recessive model) increased the risk of presenting mixed hyperlipidemia by more than 8-fold.

In general, the findings of increased HDL-C and TG are consistent with the function of the* APOA5* p.19W variant because reduced secretion of the protein decreases LPL activity [7], increasing TG levels in plasma. Moreover, APOAV is a component of HDL-C, and APOAV deficiency may reduce the metabolism of these particles, which increases their blood concentration.

*APOB p.E4181K*. APOB is the primary protein found in VLDL-C, IDL-C, and LDL-C; thus, it is important for cholesterol homeostasis. The* APOB* p.E4181K polymorphism is located in the proximal portion of the terminal carboxyl and increases LDL-C catabolism, with a consequent decrease of LDL-C and APOB levels [10]. In the present study, the p.4181K allele was associated with lower TG levels in normolipemic HT patients; this association has not been observed in other populations. The association between low TC and LDL-C levels and the p.4181K allele has been demonstrated in most populations studied to date. However, the literature is inconsistent regarding whether the p.4181K allele is protective or is a risk factor for cardiovascular diseases, as both models have been observed [10]. In this work we found a risk of developing hypertension conferred by p.4181K allele, with an OR = 1.85; 95% CI: 1.2–2.9; *P* = 0.01, which is similar to the findings of a meta-analysis of 30 case-control reports where the risk of developing cardiovascular disease and myocardial infarction was evaluated, and the authors obtained an OR of 1.73 (95% CI: 1.19–2.50) [29]. Thus, we suggest that the p.4181K allele increases the risk of developing cardiovascular disease, myocardial infarction, and hypertension; however, its pathway of action must be different from that related to lipids because this allele is associated with lower TG, TC, and LDL-C concentrations.

*FABP2 p.A55T*. The* FABP2 *p.A55T polymorphism has been extensively studied and referenced in the literature. The p.55T allele results in a higher affinity of this protein for long-chain fatty acids and, hence, greater absorption of such fatty acids [11]. In this study, the p.55T allele was associated with increased SBP in normolipemic HT patients, which differs from the findings of de Luis, who observed a decrease in SBP in nondiabetic obese subjects [30]. However, an association between the mutated allele and an increase in TG was observed in the HT patients and hyperlipemic subgroup and has been reported previously [31, 32]. This association is consistent with the increase in fatty acid absorption found in individuals with the p.55T allele and therefore enhances TG-rich lipoprotein formation [11]. Moreover, the studied groups in which this association had been observed also exhibit low HDL-C, as found in our patients with hypoalphalipoproteinemia.

*LDLR c.1959C>T*. The c.1959T allele was associated with low TG levels in HT patients and hyperlipemic subgroup, in a multiple linear regression model (Table 4). In general population of China, this allele has been associated with lower TC, LDL-C, and TG concentrations [12, 13].

*LIPC −514 C>T*. One of the primary functions of hepatic lipase is to hydrolyze TG and phospholipids, and hepatic lipase is an important enzyme in HDL-C metabolism [14]. HT patients group with the −514T allele exhibited higher SBP values compared with those carrying the −514C allele. To our knowledge, this is the first study to analyze this polymorphism in HT patients and to assess its association with blood pressure.

*LPL p.S474X.* The primary function of LPL is to hydrolyze TG from CM and VLDL-C. It has been shown that the p.474X allele increases LPL enzymatic activity; hence, it has been associated with decreased plasma TG and increased HDL-C [15]. In the present work, in the HT and HT hyperlipemic groups, the p.474X allele was associated with lower TC levels, as previously observed in a meta-analysis. The mutated allele has also been associated with lower TG and SBP levels as well as higher HDL-C levels [33]. Consistent with such results, the presence of the p.474X allele has shown in previous reports a lower risk for developing coronary heart disease [33] and hypertension (OR 0.78; 95% CI: 0.62–0.98, *P* = 0.03) [34].

*MTTP −493 G>T*. The MTTP enzyme is involved in the assembly and secretion of VLDL-C, which transports TG, cholesterol, and phospholipids [16]. In this study, the −493T allele was associated with increased SBP in HT hyperlipemic subgroup, which has not been previously reported in the literature. However, in healthy males and hypercholesterolemia patients, the mutated allele has been associated with low TC [16] and TG levels [35], respectively.

## 5. Conclusions

We highlight three important conclusions. (i) The* APOB* p.E4181K polymorphism (under the dominant model) is associated with an increased risk for hypertension. (ii) Three polymorphisms were found to be associated with systolic blood pressure levels in HT patients. Increased SBP was associated with the* FABP2 *p.55T,* LIPC *−514T, and* MTTP* −493T alleles. (iii) Modifications of the four different lipids studied herein were observed to be correlated with certain polymorphisms. Total cholesterol is decreased in subjects with the* LPL *p.474X allele; LDL-C is decreased with* FABP2* p.55T; HDL-C is increased with* APOA5* p.19W; and triglycerides are increased with* APOA5* p.19W and* FABP2* p.55T but decreased with* APOB* p.4181K and* LDLR* c.1959T. These findings indicate that polymorphisms of lipid metabolism genes modify systolic blood pressure and lipid levels and may be important for the development of essential hypertension and dyslipidemia in Mexican HT patients.

## Figures and Tables

**Table 1:** Frequencies of genotypic and allelic polymorphisms in hypertensive and normotensive subjects.

	*APOA5* p.S19W			*APOB* p.E4181K			*FABP2* p.A55T			*LDLR* c.1959C>T			*LIPC* −514C>T			*LPL* p.S474X			*MTTP* −493 G>T		

HT	*n* = 160		%	*n* = 160		%	*n* = 158^a^		%	*n* = 160		%	*n* = 154^a^		%	*n* = 160		%	*n* = 158^a^		%

Genotype	SS	97	60.6	EE	88	55.0	AA	57	36.1	CC	36	22.5	CC	38	24.7	SS	142	88.8	GG	106	67.1
	SW	56	35.0	EK	67	41.9	AT	47	29.7	CT	73	45.6	CT	67	43.5	SX	17	10.6	GT	48	30.4
	WW	7	4.4	KK	5	3.1	TT	54	34.2	TT	51	31.9	TT	49	31.8	XX	1	0.6	TT	4	2.5

	*n* = 320		%	*n* = 320		%	*n* = 316		%	*n* = 320		%	*n* = 308		%	*n* = 320		%	*n* = 316		%

Allele	S	250	77.1	E	243	75.9	A	161	50.9	C	145	45.3	C	143	46.4	S	301	94.1	G	260	82.3
	W	70	22.9	K	77	24.1	T	155	49.1	T	175	54.7	T	165	53.6	X	19	5.9	T	56	17.7

NT	*n* = 160		%	*n* = 160		%	*n* = 159^a^		%	*n* = 160		%	*n* = 157^a^		%	*n* = 159^a^		%	*n* = 159^a^		%

Genotype	SS	100	62.5	EE	111	69.4	AA	71	44.7	CC	34	21.2	CC	35	22.3	SS	135	84.9	GG	112	70.4
	SW	53	33.1	EK	42	26.3	AT	35	22.0	CT	82	51.3	CT	78	49.7	SX	22	13.8	GT	42	26.4
	WW	7	4.4	KK	7	4.3	TT	53	33.3	TT	44	27.5	TT	44	28.0	XX	2	1.3	TT	5	3.2

	*n* = 320		%	*n* = 320		%	*n* = 318		%	*n* = 320		%	*n* = 314		%	*n* = 318		%	*n* = 318		%

Allele	S	253	79.1	E	264	82.5	A	177	55.7	C	150	46.9	C	148	47.1	S	292	91.8	G	266	83.6
	W	67	20.9	K	56	17.5	T	141	44.3	T	170	53.1	T	166	52.9	X	26	8.2	T	52	16.4

*P* genotype	0.93			0.01			0.19			0.58			0.52			0.77			0.82		

*P* allele	0.85			0.04			0.23			0.69			0.86			0.27			0.65		

^a^Due to a lack of DNA, these samples were not screened. HT: hypertensive patients. NT: normotensive subjects.

**Table 2:** Distribution of dyslipidemias in hypertensive patients.

	Without	%	With	%
^ a^Hypercholesterolemia	66	73.3	24	26.7
^ b^LDL-C ≥130 mg/dL	51	56.7	39	43.3
^ c^Hypertriglyceridemia	81	90.0	9	10.0
^ d^Mixed hyperlipidemia	83	92.2	7	7.8
^ e^Hypoalphalipoproteinemia	63	70.0	27	30.0
^ f^Dyslipidemia	50	55.6	40	44.4

^a^Total cholesterol (TC) >200 mg/dL, LDL-C >130 mg/dL, and triglycerides (TG) <200 mg/dL; ^b^LDL-C ≥130 mg/dL; ^c^TC <200 mg/dL, LDL-C <130 mg/dL, and TG >200 mg/dL; ^d^TC ≥200 mg/dL, LDL-C ≥130 mg/dL, and TG ≥200 mg/dL; ^e^HDL-C <35 mg/dL; and ^f^presence of any dyslipidemia (hypercholesterolemia or hypertriglyceridemia or mixed hyperlipidemia).

**Table 3:** Results of ANOVA, Student's *t*, and correlation tests between polymorphisms and quantitative variables in normolipemic and hyperlipemic hypertensive patients.

*APOA5* p.S19W						

	Variable	Genotype SS	Genotype WW	*P*	Subgroup	

ANOVA	HDL-C (mg/dL)	41.6	67.0	0.000	Normolipemic	
	HDL-C (mg/dL)	49.5^SW^	67.0	0.001	Normolipemic	
	TG (mg/dL)	172.0	221.0	0.032	Hyperlipemic	

		Allele S	Allele W	*P *	**Correlation **	***P***

Student's *t*-test	HDL-C (mg/dL)	43.6	52.3	0.007	0.361	0.010
	TG (mg/dL)	183.5	235.5	0.023	0.315	0.048

*APOB* p.E4181K						

	Variable	Genotype EE	Genotype EK	*P *	Subgroups	

ANOVA	TG (mg/dL)	121.6	88.3	0.006	Normolipemic	

		Allele E	Allele K		**Correlation**	***P***

Student's *t*-test	TG (mg/dL)	114.6	83.2	0.003	−0.435	0.002

*FABP2* p.A55T						

	Variable	Genotype AA	Genotype TT	*P *	Subgroups	

ANOVA	LDL-C	170.2	135.9	0.018	Hyperlipemic	
	TG (mg/dL)	164.3	237.2	0.090^a^	Hyperlipemic	
	SBP (mm Hg)	145.0	156.0	0.082^a^	Normolipemic	

		Allele A	Allele T	*P *	**Correlation**	***P***

Student's *t*-test	LDL-C	162.2	141.5	0.018	−0.328	0.042
	TG (mg/dL)	169.2	216.5	0.006	0.382	0.017
	SBP (mm Hg)	147.1	155.0	0.007	0.346	0.026

*LPL* p.S474X						

	Variable	Genotype SS	Genotype SX	*P *	Subgroups	

ANOVA	TC (mg/dL)	236.0	189.4	0.035	Hyperlipemic	

		Allele S	Allele X	*P *	**Correlation **	***P***

Student's *t*-test	TC (mg/dL)	234.1	189.6	0.039	−0.335	0.035

*MTTP* −493 G>T						

	Variable	Genotype GG	Genotype GT	*P *	Subgroups	

ANOVA	SBP (mm Hg)	141.5	154.5	0.023	Hyperlipemic	

		Allele G	Allele T	*P *	**Correlation**	***P***

Student's *t*-test	SBP (mm Hg)	144.2	154.5	0.003	0.364	0.023

TG: triglycerides; TC: total cholesterol; SBP: systolic blood pressure; ^a^Nonsignificant *P* value, but significant in the remaining statistical tests.

**Table 4:** Multiple linear regression models observed in normolipemic and hyperlipemic hypertensive patients.

Variable	Allele	Constant	*B*	*P*	Subgroups
TC (mg/dL)	*LPL* p.474X	234.1	−44.5	0.039	Hyperlipemic

LDL-C (mg/dL)	*FABP2 *p.55T	162.2	−20.7	0.024	Hyperlipemic

HDL-C (mg/dL)	*APOA5* p.19W	43.6	8.7	0.007	Normolipemic

TG (mg/dL)	*APOA5* p.19W	183.5	52.0	0.023	Hyperlipemic
	*APOB* p.4181K	114.6	−31.4	0.003	Normolipemic
	*FABP2* p.55T	169.2	47.3	0.010	Hyperlipemic
	*FABP2 *p.55T	142.4	36.5	0.002	Hypertensive
	*LDLR *c.1959T		−26.3	0.024	
	*FABP2 *p.55T	190.0	53.2	0.003	Hyperlipemic
	*LDLR *c.1959T		−43.0	0.017	

SBP (mmHg)	*FABP2* p.55T	147.1	7.9	0.007	Normolipemic
	*MTTP* −493T	144.2	10.3	0.042	Hyperlipemic

TC: total cholesterol; TG: triglycerides; SBP: systolic blood pressure.
